# Insights into naturally minimised *Streptomyces albus* J1074 genome

**DOI:** 10.1186/1471-2164-15-97

**Published:** 2014-02-05

**Authors:** Nestor Zaburannyi, Mariia Rabyk, Bohdan Ostash, Victor Fedorenko, Andriy Luzhetskyy

**Affiliations:** 1 Helmholtz-Institute for Pharmaceutical Research Saarland, Saarland University Campus, Building C2.3, 66123 Saarbrücken, Germany; 2 Department Faculty of Biology, Ivan Franko National University of Lviv, Hrushevskogo str. 4, Lviv79005, Ukraine

## Abstract

**Background:**

The *Streptomyces albus* J1074 strain is one of the most widely used chassis for the heterologous production of bioactive natural products. The fast growth and an efficient genetic system make this strain an attractive model for expressing cryptic biosynthetic pathways to aid drug discovery.

**Results:**

To improve its capabilities for the heterologous expression of biosynthetic gene clusters, the complete genomic sequence of *S. albus* J1074 was obtained. With a size of 6,841,649 bp, coding for 5,832 genes, its genome is the smallest within the genus streptomycetes. Genome analysis revealed a strong tendency to reduce the number of genetic duplicates. The whole transcriptomes were sequenced at different time points to identify the early metabolic switch from the exponential to the stationary phase in *S. albus* J1074.

**Conclusions:**

*S. albus* J1074 carries the smallest genome among the completely sequenced species of the genus *Streptomyces*. The detailed genome and transcriptome analysis discloses its capability to serve as a premium host for the heterologous production of natural products. Moreover, the genome revealed 22 additional putative secondary metabolite gene clusters that reinforce the strain’s potential for natural product synthesis.

## Background

Recent advances in whole-genome sequencing have revealed that actinomycetes carry approximately 30 biosynthetic gene clusters and thus have huge potential to produce natural products. However, in practice, the majority of the biosynthetic gene clusters remain silent under standard laboratory conditions. Therefore, the main challenge in the field is to access the hidden biosynthetic potential of Actinobacteria. One approach is to clone the gene cluster on a cosmid or BAC, redesign it and then express it in a well characterised bacterial host. While identification and cloning of the gene clusters is rather straightforward, successfully expressing them in heterologous hosts remains challenging.

*S. albus* J1074 has long been known as a suitable host for the heterologous production of versatile secondary metabolites, ranging from marine *Micromonospora* secondary metabolites [1] to potent anticancer agents [2]. For example, this strain was used to express steffimycin biosynthetic genes [3], as well as fredericamycin [4], isomigrastatin [5], napyradiomycin [6], cyclooctatin [7], thiocoraline [1], and moenomycin [8] biosynthetic gene clusters. *S. albus* J1074 has a valine- and isoleucine-auxotrophic phenotype and is defective in the *Sal*I (*Sal*GI) restriction-modification system, which allows it to be genetically manipulated in a straightforward fashion. Its complete genomic sequence highlighted its naturally minimised size but also provided new directions for *S. albus* applications.

Recent attempts to construct and improve a model host for the heterologous expression of genes encoding secondary metabolites have done so by deleting nonessential genes [9,10]. However, the constructed *S. avermitilis* strain still possesses considerably larger chromosome than that of *S. albus* J1074. Genomic information can provide us with additional possibilities for optimising a given strain for heterologous production and to develop methods for the activation of otherwise silent clusters. We present the complete sequence of the *S. albus* J1074 genome and compare it to other streptomycetes whose genomes have been sequenced. Moreover, detailed transcriptome time series of 12, 36 and 60 hours of shake-flask cultures of *S. albus* J1074 have been used to profile gene expression.

## Results and discussion

### General features of the *S. albus* J1074 genome

At 6,841,649 bp, *S. albus* is one of the smallest *Streptomyces* genomes, along with *S. cattleya*; however, the latter also contains a megaplasmid pSCAT (1,809,491 bp). The genome size is an interesting feature of streptomycetes biology, and the availability of its complete genomic sequence made it possible for us to attempt to explain this phenomenon. Deep analysis of chromosomal genes has shown that *S. albus* tends to reduce the number of orthologous groups of genes. It has also the highest known GC content (73.3%) of streptomycetes. The main features of the single chromosome sequence are shown in Table 1. Unlike those of other streptomycetes genomes, the single chromosome includes seven rRNA operons (16S-23S-5S) and 66 tRNA genes (41 species). The presence of seven rRNA operons might explain the exceptionally fast growth rate and versatility of this strain [11].

**Table 1:** General features of the chromosome

**Property**	**Value**
Topology	Linear
Total size	6 841 649
Terminal inverted repeats	2 × 30 000 bp
G + C content	73.3%
Coding sequences	5832
Average gene length	1011 bp
Coding density	86.8%
Ribosomal RNAs	7 × (16S–23S–5S)
Transfer RNAs	66 (41 species)

**Figure 1:**
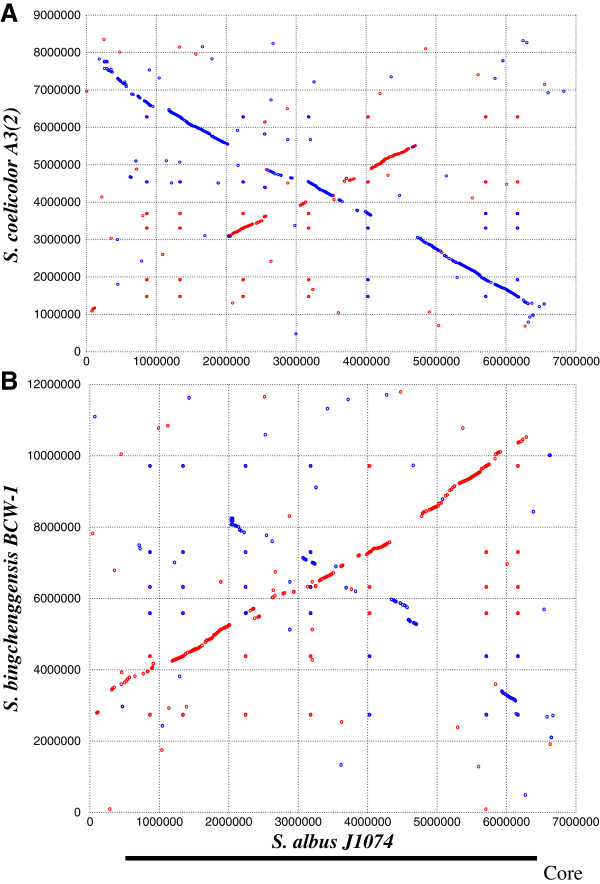
**Genomic sequence comparison of three *Streptomyces *strains. (A)***S. albus* versus *S. coelicolor*; **(B)***S. albus* versus *S. bingchenggensis* were generated with NUCmer using default settings. Matches on the same strand are in red, and those on the opposite strand are in blue. The black bar at the bottom denotes the core region, which for *S. albus* contains almost the entire chromosome.

The chromosome of *S. albus* J1074 contains 5832 predicted protein coding sequences (CDS). Of these CDS, 4665 (80%) could be could be ascribed putative functions, while the remaining 1172 ORFs (20%) were annotated as genes that code for hypothetical proteins. The origin of replication showed perfect symmetry and is situated exactly in the middle of the chromosome, located at 580 bp left of the centre, at 3 419 111–3 420 244 bp – this region contains 19 tandem DnaA box-like sequences and is flanked by the *dnaA* and *dnaN* genes. The central “core” that contains essential genes comprises nearly the whole chromosome from approximately 0.3 Mb to 6.4 Mb, while the “arms” were much smaller in comparison to those of *S. coelicolor*, with lengths of approximately 0.3 Mb (left arm) and 0.4 Mb (right arm). Therefore, its genomic topology is quite minimal compared to other sequenced actinomycetes genomes (Figure 1).

### Plasticity and receptivity

**Figure 2:**
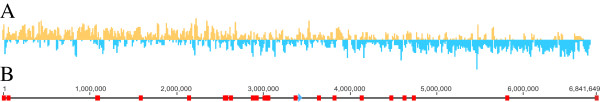
**Features of linear *S. albus *J1074 chromosome. (A)** GC-skew pattern of *S. albus* J1074 chromosome showing overrepresentation of C over G (yellow) and G over C (blue) in the strand analysed; **(B)** Distribution of mobile elements though the *S. albus* chromosome. The origin of replication is marked with a blue triangle.

Putative transposase genes are found throughout the chromosome in intact, truncated and frameshifted forms. Unlike *S. coelicolor*, in which transposases are concentrated on arms (in particular at the sub-TIR regions), virtually all insertion elements in *S. albus* are found in the core region (Figure 2). As such, the sheer distribution of mobile elements could be indicative of recent genomic perturbations. Of the 40 predicted transposase coding sequences, 17 form simple insertion elements, while the remainder are not bounded by inverted repeats. Most of them fall into 2 families, such as IS112- and IS1647-like elements. Notably, 30 putative transposase genes lie to the left of *oriC* and correlate with greater variation in GC-content DNA composition in the left half of the chromosome (Figure 2). A high degree of horizontal gene transfer can be observed 370 kb left of *oriC* (approximately 40 kb size), which is a region containing below average GC-content and multiple insertions of mobile elements.

As previously demonstrated [12,13], one of the IS112 insertion elements disrupted the gene for the restriction enzyme *Sal*I. We also identified that another IS112 element is inserted into the gene of DNA methyltransferase subunit of the Type I restriction-modification system. In addition, *S. albus* has only three restriction endonucleases and four site-specific methyltransferases. Interestingly, *S. albus* lacks the *dndA-E* operon involved in DNA phosphothiolation (variety of R/M-system) present in *S. lividans* TK24 [14,15], which explains why the given strain does not prevent incoming DNA from adding to exceptionally high transfer rates.

### Establishing strain ancestry

The taxonomic position of *S. albus* J1074 within the *S. albus* clade was obscure. First mention of this strain occurred in 1980 [11], in which J1074 was referred to as a *Sal*I system-deficient strain derived from *S. albus* G. Although, the origin of *S. albus* G is also unknown, it was used as one of *S. albus* strains in 1970 [16] to analyse the LL-diaminopinielic acid containing peptidoglycans of streptomycetes. Therefore, the interesting results of the initial attempts to study the *S. albus* J1074 genome encouraged us to clarify the strain’s taxonomic position. The sequences of the 16S rRNA genes from all *S. albus* strains available in GenBank database (Additional file 1: Table S1) were compared. According to our analysis, *S. albus* J1074 falls into one clade with strains *S. albus* subsp. albus NBRC 3422, NBRC 3711 and *S. albus* DSM 40890. Most other strains of *S. albus* subsp. albus cluster very closely in one clade and share 100% sequence similarity with only one exception – DSM 40313 (Additional file 2: Figure S1).

### Comparative overview

**Figure 3:**
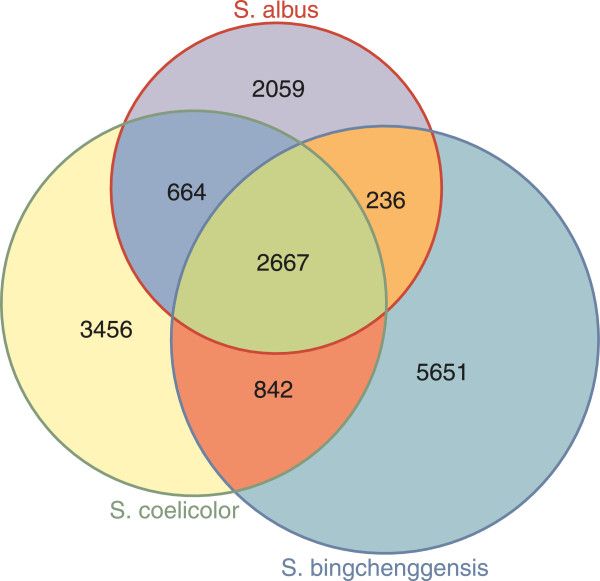
**BLASTCLUST classification of proteins into clusters.** A total of 5851 *S. albus*, 7768 *S. coelicolor*, and 10022 *S. bingchenggensis* proteins were classified. The number of shared and unique clusters, not proteins, is shown.

We compared the chromosomes of three *Streptomyces* species, namely *S. albus*, *S. coelicolor* A3(2) [17], and *S. bingchenggensis*[18] (largest sequenced *Streptomyces* to date), in order to establish the loss of regions and functions through the evolution of J1074. Dot plots generated via NUCmer software clearly demonstrated the existence of a highly conserved internal core region of each chromosome even when several inversions were found (Figure 1). Relative to the *S. bingchenggensis* BCW-1 genome, *S. albus* J1074 lacks 4.5 Mb on its chromosomal arms. We clustered *S. albus* J1074, *S. coelicolor* A3(2), and *S. bingchenggensis* BCW-1 proteins using the BLASTCLUST program with a threshold of 60% identity plus 70% length coverage (Figure 3). As such, 2811 *S. albus* J1074 proteins (48% of the total proteins), 2947 *S. coelicolor* A3(2) proteins (38%), and 2988 *S. bingchenggensis* BCW-1 proteins (30%) were classified into 2667 clusters that are commonly present in these three species. We also found 842 clusters that are absent in *S. albus* but present in both *S. coelicolor* A3(2) and *S. bingchenggensis*.

*S. albus* lacks the *whiE* gene cluster (*SCO5320* to *SCO5214*), which is involved in the biosynthesis of an aromatic-polyketide spore pigment [19]. Additionally, we found that the *bldK* genes (*SCO5112* to *SCO5116*), which encode a peptide transporter involved in morphological development in *S. coelicolor* A3(2) [20], are not present in *S. albus*. However, *S. albus* contains multiple other peptide transporter systems, one of which may function as the BldK system.

*Streptomyces* sp. linear plasmids and linear chromosomes usually contain conserved terminal palindromic sequences bound to the conserved telomeric proteins Tap and Tpg, which are encoded by the *tap* and *tpg* genes, respectively [21]. However, we were not able to identify the *tpg* gene in *S. albus*. A gene-encoding Tap domain-containing protein is located on the right end of chromosome (XNR_5804) and upstream of a pseudogene of protein with DNA-binding properties. However, as in the case of *S. griseus*, these genes appear to be non-functional [22]. While Kirby et al. [23] noted that *S. albus* lacks these genes possibly due to circular chromosome, this seems not to be the case, as the only replicon it has is linear. Therefore, we assumed that *S. albus* acquired a novel pair of Tpg and Tap proteins that have yet to be identified, as it was described for multiple linear streptomycetes plasmids [24].

Another interesting feature of *S. albus* genome is the absence of the gamma-butyrolactone system. We were not able to identify genes for signal molecules biosynthesis with the exception of one gene-coding protein of the TetR family, which shows homology to gamma-butyrolactone binding protein. Taking into account the size of the *S. albus* genome, we suggest that it was lost during chromosomal rearrangements. The A-factor instability of *S. griseus* is well known and is explained by the location of the *afsA* gene in the vicinity of one end of the chromosome [25]. Therefore, due to deregulated signalling mechanisms, this strain could have acquired a genuine, permanent capability of heterologous production of secondary metabolites.

### Minimising genetic duplicates

A total of 520 genes (8.9%) are predicted to be involved in regulation. *S. albus* J1074 codes for 35 sigma factors, which is a small number relative to other streptomycetes, such as *S. coelicolor* (65) and *S. avermitilis* (60), etc. Of these 35 sigma factors, 25 are “ECF” (extra-cytoplasmic function) sigma factors, which respond to external stimuli and activate genes involved in responses to different stresses, cell-wall homeostasis and aerial mycelium development. As with other streptomycetes, *S. albus* J1074 also has abundant two-component regulatory systems. Our analysis has revealed the presence of 60 sensor kinase genes, 42 of which lie adjacent to genes encoding response regulators that form two-component systems. In addition, there are 19 orphan response regulators encoded in this genome. In comparison, the *S. coelicolor* genome encodes 67 two-component systems [26]. There are also 27 genes encoding serine/threonine protein kinases in *S. albus* genome. As the number of two-component signal transduction systems encoded by a bacterial genome usually is proportional to the size of the genome [27] and reflects the range of signals to which bacteria can respond [28], we estimate that signal transduction is one area in which *S. albus* has retained the majority of its functions (i.e., extracellular signals).

The genes encoding members of previously described regulator many families such as LysR, LacI, ROK, GntR, TetR, IclR, AraC, AsnC, ArsR, DeoR, MarR and MerR are present in the *S. albus* J1074 genome. In addition we identified 33 putative DNA-binding proteins. A total of 442 genes (7.2%) appear to be involved in transport into or out of the cell, the majority of which are ABC transporters. Among these are permeases, ion-, amino acid-, peptide- or sugar-binding transporters, or ATP-driven membrane transporters. In addition, *S. albus* J1074 has features that still allow extensive exploitation of rich media sources. A wide range of degrading enzymes, including multiple proteinases/peptidases, seven chitinases, two glucanases, two amylases and one cellulase are predicted to be secreted from the cell. Presumably, these enzymes play a key role in breaking down the heterogeneous alternative food sources in soil.

Having all the necessary features of a streptomyces genome, *S. albus* tends to exhibit minimised duplication of genes and operons. For example, *S. albus* contains one gene for chloramphenicol resistance, while *S. coelicolor* carries two genes: *clmR1* and *clmR2*. In *S. coelicolor*, two sets of genes are responsible for the biosynthesis of wall teichoic acids (WTA): SCO2589-SCO2590 and SCO2979, SCO2998 [29]. Among these, glycosyltransferases play a central role for WTA production [30], including SCO2981, SCO2982, SCO2983, SCO2997, SCO2589, SCO2590, SCO2592. *S. albus* contains only three genes for such glycosyltransferases: XNR_1871, XNR_1873 and XNR_1874, all of which are located in a single cluster.

The *S. albus* genome has also been minimised in regard to the chaplin family proteins. The chaplins are surface-active proteins that comprise two classes: short chaplins and long chaplins [31,32]. The number of short and long chaplins varies from species to species. *S. coelicolor* has three long chaplins (ChpA–C) and five short chaplins (ChpD–H). ChpC, ChpE and ChpH are a minimal set conserved among Streptomycetes [33]. *S. albus* contains orthologs of those three short chaplins, XNR_5022 (*chpE*), XNR_5152 (*chpH*) and XNR_5153 (*chpC*) and of two long chaplins, XNR_2152 (*chpA*) and XNR_2151 (*chpD*).

*S. coelicolor* carries three operons for nitrate reductase complexes, where NarG plays central role and there are three *nar* genes – SCO0216 (*narG2*), SCO4947 (*narG3*) and SCO6535 (*narG*). In contrast, *S. albus* contains only one cluster of genes for nitrate reductase, in which XNR_0412 (*narG*) codes for the putative alpha chain of nitrate reductase. Additionally, J1074 contains only one cluster of genes for gas vesicle synthesis: XNR_4422 - XNR_4431.

### Genes for antibiotic resistance

The chromosome of *S. albus* helps to explain another distinctive characteristic of its laboratory cultivation: that the bacterium’s spectrum of resistance is not as diverse relative to *S. coelicolor* (Additional file 3: Table S2). There are 17 beta-lactamase genes and 17 dioxygenases related to the bleomycin resistance proteins, 5 rRNA methyltransferases, 5 aminoglycoside acetyltransferases and 18 other genes associated with its antibiotic resistance. Detailed examination of the genome revealed that *S. albus* J1074 contains an ortholog of SCO1321 – XNR_5511 (a *tuf3* gene encoding elongation factor, TU-3, which confers complete resistance to kirromycin and GE2270A) [34]. XNR_5423 is an ortholog of RpbA (SCO1421), an RNA polymerase-binding protein that occurs in actinomycete bacteria and confers basal levels of rifampicin resistance in *S. coelicolor*[35].

Regarding chloramphenicol resistance, *S. albus* contains XNR_2375, an ortholog of CmlR1 (SCO7526), while CmlR2 is absent [36]. Genes are present for efflux proteins for daunorubicin (XNR_2457-58, XNR_4042-43), camphor (XNR_2486-87), bicyclomycin (XNR_0140), tetracycline (XNR_3352) and one putative macrolide glycosyltransferase (XNR_4394). *S. albus* contains two genes for tryptophanyl-tRNA synthetase: XNR_3910 and XNR_3513, of which the latter is an ortholog of indolmycin-resistant Trp-synthetase from *S. coelicolor*[37]. It is worth noting that the *van*-cluster involved in vancomycin resistance is absent from the *S. albus* genome.

Another interesting feature of this strain is that *S. albus* displays sensitivity to moenomycin with a survival rate of 0.001% at 1 μg/ml, while *S. coelicolor* and most streptomycetes strains are naturally resistant to this antibiotic [8]. As the major targets of moenomycin are transglycosylases involved in peptidoglycan biosynthesis, we examined the penicillin-binding proteins (PBP) genes of *S. albus* more closely and found that it contains 17 genes for PBP that show a high degree of homology to the PBP genes of *S. coelicolor*[38]. Among those identified, XNR_2983, XNR_2736, XNR_4127, and XNR_1770 belong to the PBP-A class, while 6 genes fall into the PBP-B class. The C class is comprised of 7 genes for PBP in *S. albus*. However, analysis of amino acid sequences and domain organisation of PBP-A revealed no significant differences from those in other bacteria. Moreover, transglycosylase domains of PBP from *S. albus* contain all 5 sequences required for moenomycin binding [39]. Thus, it is likely that moenomycin susceptibility is not dependent on specific PBPs but, rather, on other structural or functional changes of the cell wall biosynthesis machinery.

### Potential for production of secondary metabolites

**Figure 4:**
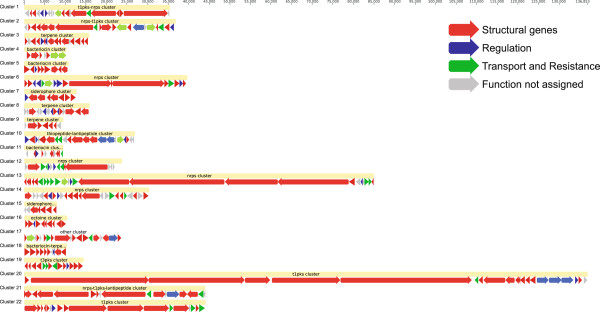
**Biosynthetic gene clusters identified in the genome of *S. albus *** **J1074.**

Genomic sequencing has revealed 22 clusters for biosynthesis of secondary metabolites (Figure 4). The distribution of these clusters is not uniform within the chromosome, as 7 clusters are located on chromosomal arms, and the remaining 15 are in the large “core” region that contains most of the essential genes. Of the 22 clusters, 4 were estimated for terpene biosynthesis, 11 for polyketides or non-ribosomal peptides, 2 for siderophores and 5 for lantibiotics and others.

Of the five terpene synthase genes, XNR_0271 and XNR_5685 are classified as phytoene synthases, while XNR_1297 is a germacradienol/geosmin synthase. Furthermore, XNR_1580 codes for terpene cyclase containing a metal binding motif and XNR_0267 encodes a putative squalene-hopene cyclase. Similar to other actinomycete strains, *S. albus* J1074 has 11 gene clusters that contain putative PKS (2), nonribosomal peptide synthetase (NRPS) (5), and PKS-NRPS hybrid genes (4). Unusually, among the few polyketide biosynthetic gene clusters, there is no type II PKS responsible for the biosynthesis of polycyclic aromatic compounds.

One of PKS1 clusters (XNR_5853-XNR_5873) is identical to gene cluster of *Streptomyces* sp. FR-008 for biosynthesis of a heptaene macrolide antibiotic FR-008/candicidin [40]. The fact that the given cluster is cryptic in *S. albus* and that the antibiotic structure is known can be used as a model for discovery of regulatory mechanisms repressing expression of gene clusters. Large non-ribosomal peptide synthetase XNR_5634 from NRPS cluster confined to the genes XNR_5613-XNR_5651 shows homology to indigoidine synthase, which is responsible for the biosynthesis of the blue pigment indigoidine. An NRPS gene cluster (XNR_0200 to XNR_0211) exhibits homology with SACTEDRAFT_2283 to SACTEDRAFT_2289 of *Streptomyces sp.* ACTE ctg00033.

### Transcription levels

Total transcriptome sequencing was performed using the strand-specific Illumina protocol, which was used to generate more than 192 million short reads. The large volume of data helped considerably in the annotation process, during which the coding sequences and their lengths were adjusted in order to not to controvert known transcript boundaries. Coding sequences in the genome represent a variety of transcription levels, with several abundant transcripts occupying the majority of the mRNA pool of the cell. Such overrepresented transcripts are exclusively of hypothetical function or are involved in the stress response. A comprehensive list of loci from *S. albus* J1074 and their respective transcription levels can be found in Additional file 4.

### Early metabolic switch

To establish whether *S. albus* J1074 is indeed outpacing other Streptomycete strains by the timing of metabolic transition to stationary growth phase, we performed strand-specific total RNA sequencing at several time points of growth in liquid TSB medium. Next, we analysed subsets of genes responsible for protein biosynthesis, phosphorus and nitrogen metabolism, morphological differentiation and sporulation.

**Figure 5:**
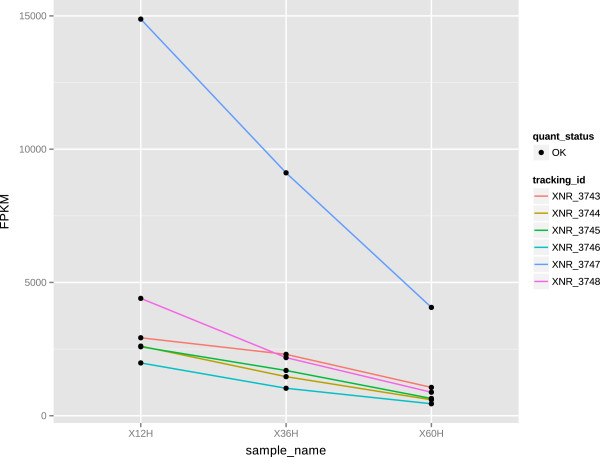
**Transcription levels of ribosomal proteins.** Transcription levels measured in FPKM of genes coding for the genes encoding ribosomal proteins S8 (XNR_3743), L6 (XNR_3744), L18 (XNR_3745), S5 (XNR_3746), L30 (XNR_3747) and L15 (XNR_3748) at 12, 36 and 60 h after culture inoculation.

**Figure 6:**
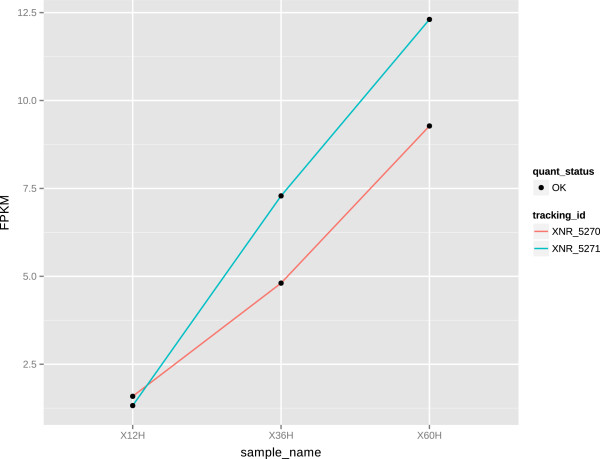
**Transcription levels of PhoPR regulatory system.** Transcription levels measured in FPKM of XNR_5270 (*phoP*) and XNR_5271 (*phoR*) genes at 12, 36 and 60 h after culture inoculation.

A subset of genes coding for ribosomal proteins and other proteins with functions in protein biosynthesis exhibited continually decreasing transcript levels during growth in the conditions tested. These genes were initially highly expressed but began to decline gradually as the cells entered the transition and stationary phases (Figure 5). The major change in expression occurred at or before 12 h from the point of inoculation, which perfectly correlates with the growth curve of *S. albus*. This point in time is regarded as a point of metabolic switch under the laboratory conditions tested. The onset of the stationary growth phase is also usually marked by a strong upregulation of the *pho*-regulon, which is controlled by the two-component kinase/regulator system of XNR_5270 (*phoP*) and XNR_5271 (*phoR*). Indeed, transcript levels of those genes began to increase as soon as phosphate was depleted from the medium (from 12 h - 36 h) (Figure 6).

The expression profiles for genes for nitrogen metabolism, and its key regulator *glnR*[41,42], also decreased after 12 h. As growth ceases, the amount of transcripts and levels of corresponding enzymes for purine, pyrimidine, and amino acid biosynthesis are reduced. The early expression of these genes is particularly surprising as nitrogen was not limiting in the medium used. Transcripts of genes that are central to nitrogen metabolism, such as XNR_1223 (GlnK), XNR_1222 (GlnD), XNR_1224 (AmtB), XNR_5568 (UreA) and XNR_4658 (GlnII) were detected in the early time points but rapidly decreased until they were nearly undetectable as cultures continued to grow.

As described for *S. coelicolor*[43], the expression profiles of genes for major glutamine synthetase (GS), GlnA (XNR_4684), NAD-specific glutamate dehydrogenase GDH (XNR_1879), and aspartate aminotransferase AspC (XNR_3703) were maintained at high levels up to the 60 h time point. While *S. coelicolor* has 5 GS-like genes, *S. albus* J1074 contains four genes for glutamine synthetase: *XNR_4684*, *XNR_4658*, *XNR_4631* and *XNR_5219*. Interestingly, transcript levels of the GS-like gene *XNR_4631* increase from the 12 h time point and amounts of *XNR_5219* became nearly undetectable after 12 h. Therefore, the rapid drop in the levels of the GlnR-regulated gene products occurred at or just before the cessation of growth (12 h). This indicates that without the demand for amino acid, purine and pyrimidine biosynthesis, the nitrogen levels in the medium become less of a limiting factor.

The expression of developmental genes increases as the cells prepare for differentiation during a metabolic switch. The expression of *whiA* is stable from 12 h to 60 h, while *whiB* levels off gradually after 12 h. Both *whiA* and *whiB* are required for the switch from elongation to division in aerial hyphae. Gene *whiA* constitutes, together with *whiB*, a *whiG*-independent converging pathway that controls sporulation in aerial hyphae. The *whiP* gene rapidly increases in expression at 12 h and then declines as rapidly to very low levels of expression. WhiP influences the coordination of aerial hyphal extension and septation, possibly by inhibiting cell division until the correct moment [44]. The expression of *whiG*, which encodes an RNA polymerase sigma factor and is a target of BldD repression, gradually decreases from 12 to 24 h and is maintained at one level until 60 h. These data support our evidence that *S. albus* sporulates in liquid culture [45] and that this process begins approximately 12 h.

Interestingly, the transcription of all of the *chp* and *rdl* genes is activated during submerged sporulation with the peak at the 12 h and shows significant levels of expression, which implies that expression of chaplins and rodlins is an obligatory part of the sporulation program, regardless of whether it occurs on plates or in liquid culture. This was also recently demonstrated for *S. venezuale*[33]. Of note, we could not detect any transcription for gene *XNR_3803* (*whiD*). Among *bld*-genes, which play a crucial role in *Streptomyces* differentiation, the highest level of expression was shown for *XNR_2837* (*bldC*), which increased from 12 h onward. Genes such as *XNR_1132* (*bldB*), *XNR_3804* (*bldM*), *XNR_2706* (*bldG*) and *XNR_3527* (*bldN*) demonstrate that peak expression occurs near the point of metabolic switch and then gradually levels off to produce constant transcript levels until 60 h.

Transcriptome analysis showed that clusters of genes for secondary metabolites in *S. albus* J1074 are cryptic. Only clusters for ectoin biosynthesis demonstrate detectable levels of expression that increase after 12 h. Other clusters showed extremely low levels of transcription that can even decrease into the stationary (biosynthetic) growth phase.

## Conclusions

The complete genome of *S. albus* J1074 was sequenced and compared to the other completely sequenced genomes of *S. coelicolor* A3(2) and *S. bingchenggensis.* The *S. albus* genome shows an interesting trend of minimisation via deletion of gene and operon duplicates. In addition to providing new insight into genome evolution, the genomic sequence is a good starting point for further *S. albus* optimisation for biotechnological application as a host for the heterologous production of natural products. The transcriptome analysis revealed the early metabolic switch in *S. albus* correlating with the fast growth of the strain. An ordered BAC library covering the genome was constructed to permit the ready application of RedET PCR-targeted gene disruption [46] to this species. The Himar1 and Tn5 transposons, site-specific recombinases and *gusA*–based reporter system applied for this strain enable very efficient and fast genome engineering of *S. albus*[47-49]. Its fast and dispersive growth is an attractive characteristic, along with sporulation in liquid culture; these properties prompted us to present *S. albus* as a new model strain for not only heterologous expression experiments but also for investigations of fundamental actinobacterial biology issues, such as growth, morphogenesis, cell division, cell wall formation and antibiotic resistance.

## Methods

### Genome sequencing, assembly and validation

The genome was sequenced using a combination of Illumina and 454 sequencing platforms. A total of 2.6 Gb of raw data was obtained, which represents a 377-fold coverage of the genome. High-molecular-mass genomic DNA isolated from *S. albus* J1074 was used to construct small (300 bp) and large-insert (4 kb and 40 kb) random sequencing libraries. Reads were assembled into 76 contigs using MIRA software [50]. BAC library of 50–70 kb (pSmart) with 9-fold genome coverage was prepared and end-sequencing (2x500 bp) was performed to provide refined contig relationships. The paired-end information was then used to join contigs into one scaffold. Gaps were closed by primer walking using specially designed PCR primers. An estimated error rate of 1 per 100 000 bases was endued to the consensus sequence. The final assembly was confirmed by pulsed-field gel electrophoresis restriction pattern using the enzymes *Ase*I, *Bcu*I and *Mau*BI (Additional file 5: Figure S2), which have infrequent recognition sites in GC-rich DNA. A GC-skew plot was generated using DNAplotter [51] software using a window size of 20 kb.

### Data analysis and annotation

Putative protein-coding sequences were predicted using the Prodigal [52] and the Rapid Annotation Server [53]. Manual curation of all coding sequences was conducted by examining the database hits of BLASTP [54] program with KEGG [55], RefSeq [56], and CDD [57] databases and the results of analyses with FRAME PLOT [58]. In some cases, the origins of leaderless transcripts were adjusted using RNA-Seq data. The tRNA and transfer-messenger RNA genes were predicted using the tRNAscan [59] and rnammer [60], respectively. Clustering of protein families was performed with BLASTCLUST [54] with minimum 60% identity and 70% length coverage. Interproscan [61] was used to confirm domain assignments. NUCmer software was used for *Streptomyces* genome comparison [62]. Secondary metabolite gene clusters were predicted in antiSMASH [63] with additional manual curation.

### Indirect RNA-sequencing

The pre-cultures of *S. albus* were prepared by placing a single colony from TSB-agar plates into a 500-ml flask with ribs (4 ribs, Labor-Ochs, Cat. No 120500) containing 5 matte glass balls (4-mm diameter, unknown source) containing 50 ml (1.5 g TSB (Fluka Analytical, T8907-1KG) + 50 ml of distilled water) of liquid TSB.

Pre-cultures were grown for 24 h in Infors Multitron Standard shakers at 150 rpm at 28°C. Subsequently, 5 ml (10% v/v) of the pre-culture was transferred into each of the new flasks with the same amount of media, ribs and balls. To account for the additional volume, 5 ml of TSB was discarded prior to addition of the culture. The flasks were then placed back in the shaker with the same parameters and each was removed upon reaching the appropriate pre-set time point. The entire liquid content of the flask was finally poured into a 50-ml Falcon Tube and spun at 5000 rpm for 10 minutes (Hettich Universal 320 R centrifuge with a 1617 rotor yields 3270 RCF). Supernatants were discarded and the wet pellets were frozen at −80°C and stored on dry ice for library construction and sequencing in the following days.

### Sequence accession id

The nucleotide sequence of *S. albus* J1074 genome has been deposited in the GenBank database under accession number [GenBank:CP004370].

### Availability of supporting data

The data sets supporting the results of this article are included as additional files.

## Competing interest

The authors declare that they have no competing interests.

## Authors’ contributions

NZ MR performed genome assembly, finished the sequence, performed annotation, comparison, RNA-Seq studies and wrote the manuscript; BO performed disc diffusion assays and helped with manuscript writing; VF helped with valuable recommendations for this manuscript; AL proposed the study, participated in its design and coordination and helped to finish the manuscript. All authors read and approved the final manuscript.

## Supplementary Material

Additional file 1: Table S1 Ribosomal 16S genes used for the classification of the studied strain. ^a^ unpublished. Click here for file

Additional file 2: Figure S1 Phylogenetic classification of *S. albus* J1074 strain. The analysis was performed using the sequences of 16S rRNA genes and Phylogeny.fr server. Percentages at the nodes represent levels of bootstrap support from 100 re-sampled datasets. Values less than 80% are not shown. Bar equals 0.02 nucleotide substitutions per site. Click here for file

Additional file 3: Table S2 Antibiotic resistance profile of *S. albus* J1074 and of *S. coelicolor* M600 (disc diffusion assay). + No growth inhibition zone was observed after 48 h of growth in the presence of a given antibiotic disc (disc diameter – 5 mm). ± Growth inhibition zone that does not exceed 1 mm in length from the disc edge. Click here for file

Additional file 4 **Transcription levels *S. albus *** **J1074.** Microsoft Excel spreadsheet document including the observed transcription levels for 5932 loci in 12, 36 and 60 h time points. Click here for file

Additional file 5: Figure S2 Sequence verification of *S. albus* J1074 chromosome by pulsed field gel electrophoresis. Fragment lengths are: *Ase*I – 3.1, 2.1 (as one band), 0.66, 0.56, 0.29, 0,05 Mb; *Bcu*I – 0.9, 0.85, 0.67, 0.64, 0.48, 0.4, 0.36, 0.35, 0.29, 0.28, 0.27, 0.24, 0.23, 0.22, 0.2, 0.2, 0.09, 0.06, 0.05, 0.045, 0.027 Mb; *Mau*BI – 1.8, 0.9, 0.8, 0.7, 0.5, 0.5, 0.38, 0.34, 0.31, 0.28, 0.24 Mb and 58, 17, 9 Kb. Three bands below 5 kb were not detectable. Click here for file
