# Supplementary material for: Insights into naturally minimised Streptomyces albus J1074 genome
Source: BMC Genomics. 2014 Feb 5;15:97. doi: 10.1186/1471-2164-15-97 (PMC3937824; doi:10.1186/1471-2164-15-97)
Supplement: Additional file 1: Table S1 — Ribosomal 16S genes used for the classification of the studied strain. a unpublished. [file 1471-2164-15-97-S1.docx]

| Strains | Origin | 16S rRNA sequences | | |
| --- | --- | --- | --- | --- |
|  |  | Reference | Length, bp | Accession |
| *S. albus* subsp. *albus* | JCM4703 | Wang et al., 2011 | 1390 | HQ537062 |
| *S. albus* subsp. *coleimyceticus* | NBRC 13840 | Tamura^a^ | 1480 | AB184522 |
| *S. albus* subsp. *pathocidicus* | NBRC 13812 | Tamura^a^ | 1467 | NR_041152 |
| *S. albus* subsp. *albus* | DSM 40313 | Swiderski^a^ | 1499 | NR_025615 |
| *S. albus* subsp*. albus* | NRRL B-2365 | Goodfellow^a^ | 1574 | DQ026669 |
| *S. albus* subsp. *albus* | NBRC 13014 | Tamura^a^ | 1475 | AB184257 |
| *S. albus* subsp. *albus* | NBRC 3418 | Tamura^a^ | 1482 | NR_041208 |
| *S. albus* subsp. *albus* | NBRC 3710 | Tamura^a^ | 1481 | AB184781 |
| *S. albus* subsp. *albus* | NBRC 3711 | Tamura^a^ | 1478 | AB184782 |
| *S. albus* subsp. *albus* | NBRC 3422 | Tamura^a^ | 1478 | AB184773 |
| *S. albus* subsp. *albus* | NBRC 3195 | Tamura^a^ | 1479 | AB184741 |
| *S. albus* | DSM 40890 | Hain et al., 1997 | 1476 | Z76689 |
| *S. albus* J1074 |  | This study | 1515 | CP004370 |
