# Supplementary figures and images for: Insights into naturally minimised Streptomyces albus J1074 genome
Source: BMC Genomics. 2014 Feb 5;15:97. doi: 10.1186/1471-2164-15-97 (PMC3937824; doi:10.1186/1471-2164-15-97)

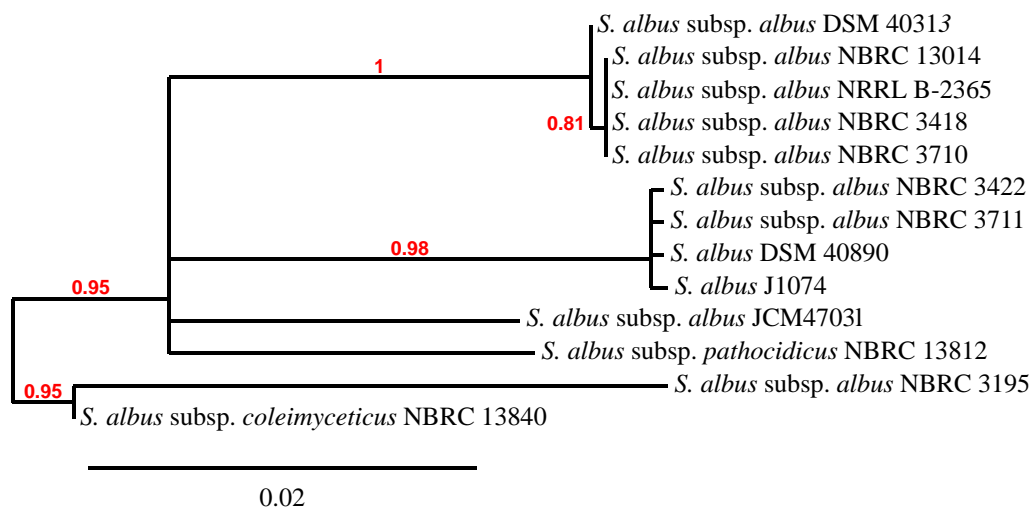

Supplement: Additional file 2: Figure S1 — Phylogenetic classification of S. albus J1074 strain. The analysis was performed using the sequences of 16S rRNA genes and Phylogeny.fr server. Percentages at the nodes represent levels of bootstrap support from 100 re-sampled datasets. Values less than 80% are not shown. Bar equals 0.02 nucleotide substitutions per site. [file 1471-2164-15-97-S2.pdf]

*AseI*

*BcuI*

*MauBI*

Ladder ( $\lambda$ -phage concatemers)

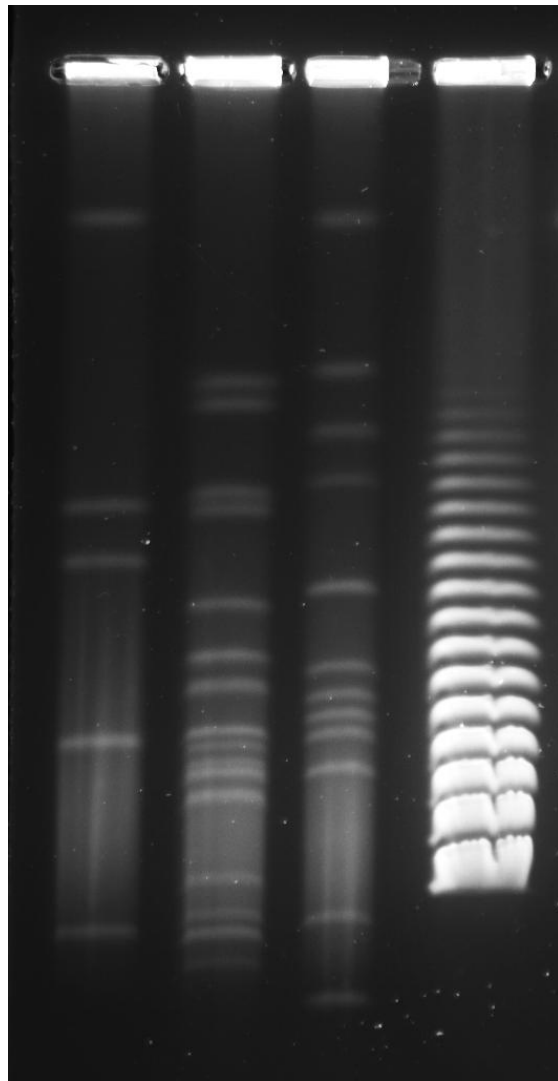

Supplement: Additional file 5: Figure S2 — Sequence verification of S. albus J1074 chromosome by pulsed field gel electrophoresis. Fragment lengths are: AseI – 3.1, 2.1 (as one band), 0.66, 0.56, 0.29, 0,05 Mb; BcuI – 0.9, 0.85, 0.67, 0.64, 0.48, 0.4, 0.36, 0.35, 0.29, 0.28, 0.27, 0.24, 0.23, 0.22, 0.2, 0.2, 0.09, 0.06, 0.05, 0.045, 0.027 Mb; MauBI – 1.8, 0.9, 0.8, 0.7, 0.5, 0.5, 0.38, 0.34, 0.31, 0.28, 0.24 Mb and 58, 17, 9 Kb. Three bands below 5 kb were not detectable. [file 1471-2164-15-97-S5.pdf]
