# Supplementary material for: Insights into naturally minimised Streptomyces albus J1074 genome
Source: BMC Genomics. 2014 Feb 5;15:97. doi: 10.1186/1471-2164-15-97 (PMC3937824; doi:10.1186/1471-2164-15-97)
Supplement: Additional file 3: Table S2 — Antibiotic resistance profile of S. albus J1074 and of S. coelicolor M600 (disc diffusion assay). + No growth inhibition zone was observed after 48 h of growth in the presence of a given antibiotic disc (disc diameter – 5 mm). ± Growth inhibition zone that does not exceed 1 mm in length from the disc edge. [file 1471-2164-15-97-S3.docx]

| Antibiotic | μg/  disc | Mode of action | J1074 | M600 |
| --- | --- | --- | --- | --- |
| A47934 | 30 | Cell wall (transpeptidation reaction) | - | ± |
| Chloroeremomycin | 30 | Cell wall (transpeptidation reaction) | - | - |
| Ristocethin | 30 | Cell wall (transpeptidation reaction) | - | - |
| Teicoplanin | 30 | Cell wall (transpeptidation and transglycosylation) | - | - |
| Enduracidin | 30 | Cell wall (transglycosylation reaction) | - | - |
| Ramoplanin | 30 | Cell wall (transglycosylation reaction) | - | - |
| Bacitracin | 10 | Cell wall and phospholipid synthesis | - | - |
| Amoxacilin | 25 | Cell wall (transpeptidation reaction) | ± | + |
| Penicillin G | 30 | Cell wall (transpeptidation reaction) | + | + |
| D-cycloserine | 30 | Inhibition of synthesis of cell wall precursors | ± | + |
| Cephradine | 30 | Cell wall (transpeptidation reaction) | - | + |
| Tunicamycin | 30 | Inhibition of synthesis of cell wall precursors | - | ± |
| Cefalexin | 30 | Cell wall (transpeptidation reaction) | - | ± |
| Fosfomycin | 30 | Inhibition of synthesis of cell wall precursors | + | + |
| Ticarcillin | 75 | Cell wall (transpeptidation reaction) | - | + |
| Polymyxin | 30 | Cell membrane | + | + |
| Streptomycin | 15 | Protein synthesis (16S rRNA of 30S subunit) | - | - |
| Thiostrepton | 50 | Protein synthesis (L11 protein of 50S subunit) | - | - |
| Rifampicin | 30 | Transcription (RNA polymerase) | - | ± |
| Novobiocin | 50 | DNA gyrase (GyrB) | - | - |
| Moenomycin A | 5 | Cell wall (peptidoglycan glycosyltransferase) | - | + |
| Landomycin D | 30 | Unknown | - | + |
| Nogalamycin | 10 | DNA intercalation | ± | - |
| Urdamycin B | 20 | DNA intercalation | - | ± |
| Coumermycin | 30 | DNA gyrase (GyrB) | - | - |
| Kanamycin | 50 | Protein synthesis (30S subunit) | - | - |
| Erythromycin | 25 | Protein synthesis (50S subunit; translocation step) | ± | + |
| Tetracycline | 10 | Protein synthesis (30S subunit; aa-tRNA entry) | - | + |
| Chloramphenicol | 35 | Protein synthesis (peptidyltransferase) | - | ± |
